# On the Action of Medicine in the System

**Published:** 1867-04

**Authors:** 


On the Action of Medicines in the System. By FREDERICK WILLIAM HEADLAND, M.D., B.A., F.L.S., Fellow of the Royal College of Physicians, etc., etc. Fifth American, from the Fourth London, Edition, revised and enlarged. Philadelphia: LINDSAY & BLAKISTON. 1867.

This new edition of a most valuable work is published in good style, on plain, fair type, and contains 431 pages. The former editions have been so long before the profession, and so extensively read, that their merits are well known. The work is worthy of a place in the library of every practicing physician. For sale by W. B. KEEN & Co., 148 Lake St. Price $3.

